# Block Copolymers with Crystallizable Blocks: Synthesis, Self-Assembly and Applications

**DOI:** 10.3390/polym14040696

**Published:** 2022-02-11

**Authors:** Holger Schmalz, Volker Abetz

**Affiliations:** 1 Macromolecular Chemistry II and Bavarian Polymer Institute, Universität Bayreuth, Universitätsstraße 30, 95440 Bayreuth, Germany; 2 Institute of Physical Chemistry, Universität Hamburg, Grindelallee 117, 20146 Hamburg, Germany; 3 Institute of Membrane Research, Helmholtz-Zentrum Hereon, Max-Planck-Straße 1, 21502 Geesthacht, Germany

## Abstract

Block copolymers with crystallizable blocks are a highly interesting class of materials owing to their unique self-assembly behaviour both in bulk and solution. This Special Issue brings together new developments in the synthesis and self-assembly of semicrystalline block copolymers and also addresses potential applications of these exciting materials.

Block copolymers bearing one or more crystallizable blocks have moved into the focus of current research owing to their unique self-assembly behaviour both in bulk and in solution. The bulk morphology and, hence, the properties of semicrystalline block copolymers are influenced by a complex interplay between crystallization and micro phase separation. Depending on the segregation strength (confinement) in the melt, crystallization can either be confined in the pre-existing microphase-separated morphology for strongly segregated melts, whereas for weakly segregated systems, a “breakout crystallization” can occur, which overwrites any existing morphology leading exclusively to lamellar structures [1,2,3,4,5,6,7,8,9]. This opens a broad parameter space for tuning the properties of semicrystalline block copolymers in bulk. First studies on semicrystalline AB diblock, ABA triblock and multiblock copolymers with one crystallizable block based on poly(ethylene oxide) (PEO), polyester blocks like poly(*ε*-caprolactone) (PCL), or polyethylene (PE, based on hydrogenated poly(1,4-butadiene)) have already been reported in the mid-1970s to 1980s [10,11,12,13,14,15,16,17]. An important milestone in this field was the development of ABC triblock terpolymers with one or two crystallizable blocks based on polystyrene-*block*-poly(1,4-butadiene)-*block*-poly(*ε*-caprolactone) (SBC) and the corresponding hydrogenated analogues with PE middle blocks (SEC), reported first by the group of *R. Stadler* in 1996 and 1998, respectively, and intensively studied thereafter together with the group of *A. J. Müller* [18,19,20,21,22]. Shortly after, in 1998, *Floudas* et al. reported on the first *µ*-ABC miktoarm star terpolymer with two crystallizable PEO and PCL blocks [23]. An important and technically highly relevant application of block copolymers with crystallizable blocks are thermoplastic elastomers. Here, ABC triblock terpolymers with a glassy polystyrene and a semicrystalline PE end block were shown to exhibit superior elastic properties compared to conventional amorphous ABA-type thermoplastic elastomers at moderate deformations [24,25]. Additionally, commercially available multiblock copolymers with semicrystalline polyamide or polyester hard blocks and polyether-based soft blocks are well-known thermoplastic elastomers and have inspired the development of more complex multiblock copolymers with improved elasticity employing well-defined ABA triblock copolymers as soft segments [26]. Some of the semicrystalline multiblock copolymers containing polyether segments, especially poly(ethylene oxide) segments, also attracted interest for gas separation membranes [27,28,29]. New synthetic concepts give access to even more complex block copolymer architectures such as triblock or tetrablock copolymers with three or even four different crystallizable blocks, [30,31] as well as to the implementation of new semicrystalline blocks, e.g., poly(vinylidene fluoride) (PVDF) [32]. Some of these recent developments are addressed in this Special Issue.

Crystallization-driven self-assembly (CDSA) of block copolymers with one core-forming, crystallizable block has developed to an extremely active and innovative field of research, starting from the first observation of defined cylindrical micelles with crystalline poly(ferrocenyl dimethylsilane) (PFS) cores in 1998 [33] and following the development of living CDSA in the groups of *I. Manners and M. A. Winnik* [34,35,36,37,38,39,40,41,42]. This paved the way to a myriad of crystalline-core micellar structures and hierarchical super-structures that were not accessible before via the self-assembly of fully amorphous block copolymers, e.g., cylindrical micelles with defined length, length distribution, and corona chemistries (block type or patchy corona), branched micelles, non-centrosym metric cylindrical micelles, and fascinating micellar superstructures (e.g., 2D lenticular platelets, scarf-shaped micelles, multidimensional micellar assemblies, cross and “wind mill”-like supermicelles). Another intriguing material class is based on amphiphilic crystalline-core micelles with poly(*L*-lactide) (P*L*LA) or corresponding stereocomplexes (P*L*LA/P*D*LA (poly(*D*-lactide)), showing interesting potential for biomedical applications, such as controlled release and drug delivery [43,44].

This Special Issue brings together new developments in the synthesis and self-assembly (bulk and solution) of block copolymers with crystallizable blocks, including emerging applications of these exciting materials. In a fundamental work, *Rahman* studied the use of semicrystalline multiblock copolymer membranes with polyether soft segments for hydrocarbon separation [45]. The permeability of hydrocarbons was found to decrease with the number of carbons and polytetrahydrofuran (PTHF)-based systems were superior to PEO-based systems in terms of permeability and permselectivity, making these systems interesting for applications in the petrochemical industry. In addition, the lower performance of multiblock copolymers with longer PEO soft segments was attributed to partial PEO crystallization. The combination of homologation (C1 polymerization) with ring-opening (ROP) or iodine transfer polymerization (ITP) is a facile route to block copolymers with polymethylene (structurally identical to PE) blocks. *Hadjichristidis and Müller* et al. utilized this approach to synthesize PE-*b*-PEO-*b*-PCL triblock terpolymers, in which all three blocks are able to crystallize. Here, PE crystallizes first upon cooling from the phase-separated melt followed by PCL and PEO [46]. They note that a combination of different characterization techniques (DSC, WAXS, PLOM) is necessary to fully deduce the complex behaviour of triple crystalline triblock terpolymers. In a joint work with *Maiz* et al. phase transitions in PE-*b*-PVDF diblock copolymers and blends were studied [47]. Due to the polymorphic nature of semicrystalline PVDF control over crystal structure is crucial, as for example the piezoelectric and ferroelectric *β*-phase is interesting for applications in electronic devices or renewable energies.Compared to PVDF homopolymer the formation of the *β*-phase was found to be strongly promoted in PE-*b*-PVDF diblock copolymers at low cooling rates. Living, stereoselective olefin polymerization is an efficient method for the synthesis of double crystalline diblock copolymers with PE and *s*PP (syndiotactic polypropylene) blocks, as described by *De Rosa* et al. [48]. By using selective crystalline substrates for the epitaxial crystallization of PE (benzoic acid) and *s*PP (*p*-terphenyl), well-ordered morphologies with crystalline lamellae of PE and *s*PP highly oriented along one direction are accessible. A relatively new approach is the evaporation-induced confinement assembly (EICA) of semicrystalline block copolymers in microemulsions that after solvent evaporation gives rise to microparticles with confinement specific morphologies, e.g., helical cylinders or axially stacked rings. In this context, *Gröschel and Schmalz* et al. have studied the confinement assembly of a series of PS-*b*-PB-*b*-P*L*LA triblock terpolymers [49]. It turned out that over a broad composition range, microparticles with predominantly hexagonally packed core–shell cylinders consisting of a P*L*LA core, a PB shell and a PS matrix were formed, which upon hydrolysis of the P*L*LA block resulted in highly porous microparticles with pronounced surface corrugations.

Considering the CDSA of block copolymers with crystallizable blocks in solution, this Special Issue includes two reviews focussing on the preparation and application of micelles with a patch-like microphase-separated (patchy) corona [50], as well as on glycine-based diblock copolypeptoids [51], respectively. Patchy micelles can be prepared by CDSA of triblock terpolymers with crystallizable middle blocks and two incompatible amorphous end blocks, or from mixtures of diblock copolymers with one common crystallizable block. Owing to their unique corona structure, patchy micelles can be utilized as highly efficient surfactants and blend compatibilizers, as nanoparticle templates, and in heterogeneous catalysis. Polypeptoids with N-substituted polyglycine backbones are a promising class of materials as, in contrast to natural polypeptides, they provide a good thermal stability, solubility in organic solvents and protease stability. This can be attributed to the absence of hydrogen bonding and stereogenic centres. Crystallinity can be easily tuned by the length of the alkyl substituents, giving rise to bioinspired worm-like 1D nanofibrils, nanorods and nanosheets. In an intriguing study, *Reiter* et al. prepared stacked lamellar crystals from a PS-*b*-PEO diblock copolymer in solution using a self-seeding approach [52]. By varying the diblock copolymer concentration and employed self-seeding temperature control over size, the number of platelet-like crystals and even the number of stacked lamellae in the crystals was achieved. A seed-trapping protocol was developed by *Guerin and Winnik* et al. to study the impact of seed fragmentation on CDSA to cylindrical micelles at elevated temperatures, where both seed dissolution and fragmentation occur [53]. Seed fragmentation was found to increase with annealing time at elevated temperatures, resulting in a decrease in length of the regrown cylindrical micelles. Furthermore, kinetics follow a stretched exponential that might indicate a fractionation upon crystallization as the rate of unimer addition to the seeds depends on the length and fraction of the crystallizable block. Finally, going toward potential applications in tissue engineering, a systematic study on the reinforcement of alginate hydrogel matrices with fibre-like micelles prepared by living CDSA of a PCL-*b*-PMMA-*b*-PDMA triblock terpolymer (PMMA = poly(methyl methacrylate); PDMA = poly(*N*,*N*-dimethyl acrylamide)) is presented by *Dove and O’Reilly* et al. [54]. Varying the micelle length and concentration in the hydrogel revealed an optimum fibre micelle length of 500 nm at a loading of 0.1 wt%, resulting in a significantly increased strain at flow of 37%.

In summary, the manuscripts in this Special Issue provide a nice overview of the recent developments in block copolymers with crystallizable blocks, spanning from synthesis to self-assembly approaches and potential applications.
